# Patellar Tendinopathy May not Be the Proper Term for Patients With Clinical Diagnosis of Patellar Tendon Disorder

**DOI:** 10.5812/traumamon.15301

**Published:** 2014-03-01

**Authors:** Dimitrios Stasinopoulos

**Affiliations:** 1 Department of Health Sciences, European University of Cyprus, Nicosia, Cyprus

**Keywords:** Patella, Tendinopathy, Diagnosis

*Dear Editor,*

Chronic patellar tendinopathy is a common clinical condition that is managed by physical therapists and is common among athletes and non-athletes alike. Patellar tendinopathy is characterized by the absence of inflammatory cells and prostaglandins and an increased presence of fibroblasts and disorganized collagen (1). Therefore, this condition is not inflammatory as physicians had thought but is a degenerative condition. Extrinsic factors such as inappropriate footwear, sport technique, training errors, and intrinsic factors such as muscle weakness and/or inflexibility, and misalignment are the main factors that lead to patellar tendinopathy (1-3). Functional activities such as squat or hop can cause pain in this condition (2, 3). The ideal term for clinical diagnosis is patellar tendinopathy because this term refers to the painful tendon without implying the pathology (1). Jumper’s knee was the first diagnostic term used for this condition. However, this condition can occur in people who are not athletes and athletes who do not perform jumping in their sports. Thus, jumper’s knee is not an appropriate term for clinical diagnosis (2). Patellar tendonitis is also an incorrect term for clinical diagnosis because the condition is not inflammatory but is degenerative as mentioned. The best diagnostic term may be the term patellar tendinosis because this term refers to the pathology of the tendon (1-3). The most common site of pain in patellar tendinopathy is the inferior pole of the patella. Patellar tendinopathy pain can also be reported at the tibial tuberosity, at the superior pole of the patella and at the midportion of the tendon (1-3). There are four sites of pain in this condition, and an appropriate diagnostic term is needed for each specific pain site. Therefore, the term patellar tendinopathy is a general diagnostic term and cannot be used for all the above-mentioned sites of pain. Moreover, it is unknown if the pathology of the above-mentioned sites are the same. Many physiotherapy techniques have been recommended for the treatment/rehabilitation of patellar tendinopathy such as electrotherapeutic (ultrasound, ESWT, laser, iontophoresis) and non-electrotherapeutic modalities (exercise programs, soft tissue manipulation, and acupuncture). All the above-mentioned treatments intend to improve symptoms (pain and function) of patellar tendinopathy but have totally different mechanisms of action. However, a treatment is effective when it reverses the pathology of the tendinopathy and not only improve the symptoms. Nowadays, eccentric training is the most common physiotherapy treatment for patients with patellar tendinopathy (1-3). The question that arises is whether eccentric training is effective for all sites of patellar tendinopathy? For example, eccentric training with dorsiflexion is effective for patients with mid-portion Achilles tendinopathy (4-9), but eccentric training without dorsiflexion has positive effects on patients with insertional Achilles tendinopathy (10). Therefore, different sites of Achilles tendinopathy are managed with different protocols of eccentric exercises. Eccentric training consisting of squats is an effective treatment approach when the patellar tendinopathy is at the inferior pole of the patella; however, no studies have investigated the effectiveness of eccentric training on other sites of patellar tendinopathy. Thus, studies determining the effectiveness of eccentric exercises at other sites of patellar tendinopathy are needed. Future well-designed studies are needed to find firstly, the most appropriate diagnostic term for each site of patellar tendinopathy and secondly, the appropriate protocol of eccentric training for each site of pain and the pathology of this disorder.
